# The Significance of Lactoperoxidase System in Oral Health: Application and Efficacy in Oral Hygiene Products

**DOI:** 10.3390/ijms20061443

**Published:** 2019-03-21

**Authors:** Marcin Magacz, Karolina Kędziora, Jacek Sapa, Wirginia Krzyściak

**Affiliations:** 1 Department of Medical Diagnostics, Faculty of Pharmacy, Jagiellonian University Medical College, Medyczna 9, 30-688 Kraków, Poland; marcin.magacz@gmail.com (M.M.); nitrogen.com@gmail.com (K.K.); 2 Department of Pharmacodynamics, Faculty of Pharmacy, Jagiellonian University Medical College, Medyczna 9, 30-688 Kraków, Poland; jacek.sapa@uj.edu.pl

**Keywords:** lactoperoxidase, caries prophylaxis, periodontitis prophylaxis, saliva, dentifrice

## Abstract

Lactoperoxidase (LPO) present in saliva are an important element of the nonspecific immune response involved in maintaining oral health. The main role of this enzyme is to oxidize salivary thiocyanate ions (SCN^−^) in the presence of hydrogen peroxide (H_2_O_2_) to products that exhibit antimicrobial activity. LPO derived from bovine milk has found an application in food, cosmetics, and medical industries due to its structural and functional similarity to the human enzyme. Oral hygiene products enriched with the LPO system constitute an alternative to the classic fluoride caries prophylaxis. This review describes the physiological role of human salivary lactoperoxidase and compares the results of clinical trials and in vitro studies of LPO alone and complex dentifrices enriched with bovine LPO. The role of reactivators and inhibitors of LPO is discussed together with the possibility of using nanoparticles to increase the stabilization and activity of this enzyme.

## 1. Introduction

Lactoperoxidase (LPO, EC.1.11.1.7) is an enzyme secreted, inter alia, to saliva, milk, and other body fluids, and participates in an unspecific humoral immune response directed against bacteria, fungi, and viruses within mucous membranes [1]. LPO forms the LPO system together with thiocyanate ions or iodides or bromides and hydrogen peroxide. The mechanism of action of this system is based on oxidation of thiocyanate ions (also iodides and bromides) to hypothiocyanite ions (hypoiodides and hypobromides) with the use of hydrogen peroxide. Hypothiocyanite ions oxidize the thiol groups of amino acid residues of microbial proteins, leading to their impaired function and, thus, inhibition of the cell division or death of the microorganism [2].

In the context of the oral cavity, the LPO system is one of the main mechanisms of anti-caries defense [3], moreover, regulating the composition of microflora and preventing the growth of pathogenic microorganisms present in periodontitis [4]. The physiological properties of the enzyme have been used in the prevention of these diseases through the creation of oral hygiene preparations enriched with the LPO system. The industrial source of this enzyme is bovine milk, whose methods of purification have been well defined, resulting in an enzyme characterized by large structural and functional similarity to human lactoperoxidase [5].

The aim of this review was to present the latest knowledge about the physiological role of human salivary lactoperoxidase. In addition, the paper presents the results of clinical trials of dentifrices enriched with LPO and in vitro tests of the LPO system itself. The role of reactivators and inhibitors of LPO, which have been intensively investigated in recent years, has also been described together with the use of nanoparticles to stabilize and increase the enzyme activity.

## 2. Genetics, Structure and Physicochemical Properties of Lactoperoxidase

### 2.1. Genetics

Lactoperoxidase together with myeloperoxidase (MPO), thyroid peroxidase (TPO), and eosinophil peroxidase (EPO), belong to the family of animal heme peroxidases, i.e., one of the four superfamilies of heme peroxidases [6]. Genes encoding human LPO, MPO, and EPO are found on chromosome 17 and form a cluster covering a region of 90,000 base pairs [7]. Their proximity of location and the same number of exons (12) may suggest that they evolved from one ancestral gene which has been duplicated [8]. In cattle, the gene of LPO is located on chromosome 19 and has 18 exons [9].

Comparison of cDNA sequences of cloned human milk LPO [10] with a sequence of cloned salivary peroxidase (SPO) [11] and other sequences of human LPO [7,12], showed sequence identity, which means that lactoperoxidase contained in saliva and other body fluids is a product of the same gene found on chromosome 17q22 [7].

In humans, there are three possible transcript variants created as a result of the alternative splicing of LPO mRNA, i.e., a major variant 1 (V1), including the complete coding sequence, variant 2 (V2), lacking exon 3, and variant 3 (V3), lacking exon 3 and 4. This is associated with the termination of translation after the secretory signal peptide in the case of V2, and a propeptide deletion in the case of V3. It has been shown that both V1 (the most numerous one) and V3 have enzymatic activity, and the activity of V3 is greater than that of V1 [13].

### 2.2. Structure and Physicochemical Properties

Lactoperoxidase (both human and bovine) is a single chain, monomeric glycopeptide of approximate mass of 80,000 Da (human) or 78,000 Da (bovine) [5,14]. Each LPO molecule contains one molecule of modified autocatalytic heme B in its active center [9,15], 

Like in many peroxidases, the calcium atom associated with Asp227 plays an important role in maintaining structure, thermal stability, and enzyme activity. Shin et al. showed that modification of this protein leading to the loss of calcium binding capacity results in a complete loss of activity [16], while Booth et al. described the consecutive precipitation of the enzyme by its subjecting to guanidinium hydrochloride to release calcium [17].

The mRNA analysis showed that both human and bovine LPO is composed of 712 amino acid residues and is characterized by 83% similarity, while protein analysis showed that human LPO is composed of 632 amino acid residues (including 16 cysteine residues) and a bovine one of 612 (including 15 cysteine residues) [9,15,18]. The difference between the theoretical length of the chain and the length of the analyzed protein results from the occurrence of post-translational modifications consisting of the cleavage of the propeptide and the signal peptide [13]. Bovine LPO can undergo *N*-glycosylation in five positions, while human LPO can undergo *N*-glycosylation in four [14]. During the SDS-PAGE analysis of human LPO, one can identify several bands derived from this enzyme. This fact can be explained by the presence of LPO with different degrees of glycosylation [16,19]; although this phenomenon may also be related to the heterogeneity of the protein chain [10].

Differences in structure, physicochemical properties and chemical activity between human and bovine lactoperoxidase are small (Table 1). Due to their high similarity and the lower price of bovine LPO, scientific research often uses it, as its purification procedures have also been well described. For industrial applications, the most commonly used enzyme is LPO from bovine milk [10].

## 3. Secretion in Physiological and Pathological Conditions

Lactoperoxidase is secreted to milk [16], tears [21], cervical mucus [22], mucus of the respiratory tract [23], and saliva (by epithelial cells of the acinus in submandibular and parotid salivary glands) [24,25]. In addition to epithelial lactoperoxidase, saliva contains myeloperoxidase derived from azurophilic granules of polymorphonuclear leukocytes, whose concentration increases with the appearance of dentition [26]. The increase in MPO concentration is associated with oral inflammation and is characteristic of periodontitis [27,28]. Unlike myeloperoxidase, the secretion of epithelial LPO is not dependent on the occurrence of inflammation. Infammation, however, does have an effect on function of LPO. Intensification of oxidation processes increases the prodution of H2O2 which, in turn, directs LPO into the peroxidation cycle which is inefficient in terms of formation of reactive products [29].

After the secretion to saliva, part of salivary peroxidase undergoes irreversible adsorption on the tooth enamel surface [30], dental plaque [31], and synthetic surfaces [32], which is reinforced by its defensive properties against microbial colonization of these surfaces. In addition, the adsorption process may contribute to an increase in the specific activity of the enzyme [33]. A strong modulating effect on this phenomenon has been demonstrated by other salivary proteins, mainly mucins [34].

The concentration of salivary lactoperoxidase undergoes changes throughout life. Its secretion is identified in neonates a few hours after delivery, while its concentration in this group shows high inter- and intraindividual variability [35]. Salvolini et al. showed that the highest concentrations of LPO in saliva are met in a group of healthy individuals aged 10–24, then its concentration decreases with age [36]. Changes in the secretion of LPO due to the circadian cycle have also been identified, with the highest concentration occurring around noon [37], which may be associated with increased physical activity [38]. During exercise, it comes to the formation of superoxide anion radical in the mitochondria of skeletal and cardiac muscles, which is then spontaneously or catalytically (with superoxide dismutase) converted to H_2_O_2_. In addition, there is an increase in the activity of NAD(P)H oxidase and xanthine oxidase also responsible for the formation of hydrogen peroxide [39]. It has been shown that in response to the reactive oxygen species formed during exercise, it comes to an increase in the production of salivary antioxidants, including LPO responsible for the decomposition of toxic H_2_O_2_ [40].

Salivary LPO concentration changes over the course of the menstrual cycle, and the peak of secretion overtakes ovulation by a few days [41,42]. In addition, its elevated level is observed in the third trimester of pregnancy [43]. The reason for this increase is the estrogen sensitivity of the salivary glands due to the expression of estrogen receptors β (ER β) on the acinar and ductal cells of salivary glands [44,45].

Another factor that can affect the activity of LPO in saliva is smoking and alcohol consumption. Goi et al. showed that peroxidase activity in smokers’ saliva is lower than in nonsmokers [46], which may be the result of irreversible inactivation of LPO by hydrogen cyanide present in tobacco smoke [47,48]. Waszkiewiecz et al. described significantly increased levels of salivary peroxidase in people who drink alcohol, which is not the effect of thickening caused by decreased salivary flow, but may be a response to an increased production of superoxide anion radical and hydrogen peroxide by CYP2E1 (belonging to the cytochrome P450 family) responsible for the ethanol metabolism [49]. This cytochrome is induced at the level of transcription, translation and post-translational modifications in people who consume ethanol on a long-term basis [50,51]. A different effect was observed among patients with acute alcohol intoxication where LPO level was significantly lower at 36 and 108 h after poisoning [52].

Xylitol, a polyol used in nonfluoride caries prophylaxis, besides depriving microorganisms of energy in futile cycle of 5-xylitol phosphate formation followed by its dephosphorylation and excretion [53], stimulates the secretion of LPO into the saliva [54] without affecting its adsorption on enamel [55]. Numerous studies are conducted in search of a connection between the occurrence of certain oral conditions and changes in the LPO synthesis. A decrease in its concentration has been reported in periodontitis (28 juvenile patients; 46 subjects, 25–54 years) [37,56] in contrast to patients with both type 1 diabetes and periodontitis (10 patients) who demonstrated an increase in LPO activity which may be associated with co-occurring xerostomia and production of concentrated saliva [57,58]. Other studies have shown no significant associations between the occurrence or severity of periodontitis and LPO salivary concentration [59,60]. Bielawski et al. described an increase in LPO salivary concentration in patients with caries (*n* = 27) compared to the healthy group (*n* = 8) [61], while no significant differences between these groups were found in the studies conducted by Lamberts et al. (29 patients, 29 controls) [62]. The search for the relationship between the concentration/activity of LPO is also conducted among patients with oral lichen planus [45] or aphthous stomatitis [63]. Discrepancies in the results obtained by particular teams may result from too small homogeneity of the examined groups, inadequate power of statistical tests and numerous physiological factors affecting the release of LPO.

## 4. Industrial Sources of LPO and Methods of Its Purification

**Figure 1:**
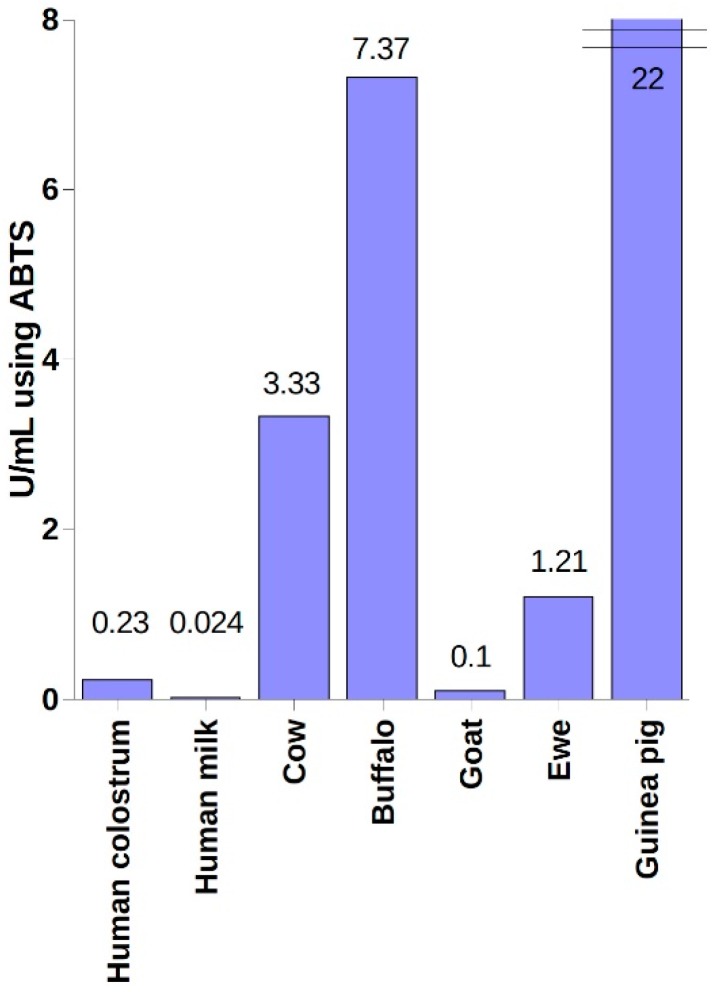
Activity of lactoperoxidase (LPO) in human milk and colostrum [35], milk of cows [64], buffalos [65], goats [66], sheep [67], and guinea pigs [69]. Comparison of LPO activity studies conducted by different teams may be problematic and subject to error due to the lack of standardization of the conditions of the analysis and the applied unit of activity.

Milk is a body fluid most rich in lactoperoxidase. This enzyme has been identified in milk in humans [35], cows [64], buffalos [65], goats [66], sheep [67], camels [68], and guinea pigs [69]. Figure 1 shows the activity of LPO in the milk of different species. Bovine milk is the most commonly used source of LPO both for laboratory and in vivo use due to its high availability and high LPO concentration of ~30 mg/L depending on the diet or time of the day or year [18]. Both pure LPO preparations and other microbiologically reactive components such as lactoferrin or immunoglobulins are used in the production of oral hygiene preparations [70,71]. There are methods that have been developed for obtaining clean preparations of LPO and lactoferrin in one process [72].

LPO purification procedures on both industrial and laboratory scales cover many stages and are time-consuming. Prior to the application of specific cleaning techniques, certain processes are used to densify the material and eliminate the main undesirable substances. These processes include fat centrifugation, removal of casein by adding rennet, removal of unnecessary major milk protein fractions, and concentration by precipitation with ammonium sulfate [5,64].

The use of affinity chromatography allows to obtain very pure LPO preparations (e.g., purification fold 3397, 7.6% yield, using IgG anti-LPO [16]), however this method is relatively expensive. Atasever et al. developed a one-step method based on sulfonamide affinity chromatography characterized by 61.3% yield and purification fold of 409 [73].

Methods based on ion exchange chromatography are preferred for a large-scale production but have lower purification fold compared to affinity chromatography methods [74]. A method using ion exchange resins (CM-cellulose) developed by Borzouee et al. obtained a LPO preparation with a 10.26% yield and purification fold of 59.13 [5]. Uguz et al. described a method using Amberlite CG 50 H^+^ ion exchange resin and double gel filtration using Sephadex G-50 and Sephadex G-100 that obtained a LPO formulation with a 28% yield and purification fold of 11.5 [64]. 

The purity of the obtained LPO preparations can be determined by direct measurement of absorbance at 412 nm (reflecting LPO hem concentration) and at 280 nm (reflecting protein concentration) as well as their ratio (R_z_ coefficient). The closer R_z_ value to 0.95 (absorbance ratio at 412 nm and 280 nm of pure LPO), the lesser LPO preparation is contaminated with other proteins [75].

## 5. Substrates—Availability and Importance

### 5.1. Hydrogen Peroxide

Hydrogen peroxide concentration in saliva equals to 8–13 μM under physiological conditions [76], and increases several fold in inflammatory diseases of the mouth [77]. Its production is ensured by both oral microorganisms as well as by the cells and enzymatic systems of the host [18], while bacterial production is sufficient to ensure the proper functioning of the salivary lactoperoxidase system.

Bacteria of Streptococcus genus, such as *S. sanguinis*, *S. mutans*, *S. sobrinus*, and *S. mitis*, belong to the main microorganisms involved in production of H_2_O_2_ [78,79,80]. Microorganisms secrete it in order to inhibit the growth of other microorganisms, regulate the biofilm formation, and facilitate the exchange of genes between bacteria in biofilm [81]. 

Bacterial production of hydrogen peroxide is most strong under aerobic conditions at the access to simple sugars and is the result of the action of enzymes such as NADH oxidase (EC.1.6.3.1) [80], lactic acid oxidase (LAo, EC.1.1.3.2) (in the stationary phase) [82], l-amino acid oxidase (EC.1.4.3.2) [83], and predominantly pyruvate oxidase (EC.1.2.3.3) (in the log phase) [84].

The ability to produce hydrogen peroxide by human salivary gland cells was proven only in the first years of the 21^st^ century. It has been shown that it is synthesized as a result of the action of NADPH oxidases (NOX 1–5 and DUOX2 dual oxidase) and superoxide dismutase (SOD, EC.1.15.1.1) [85,86].

The industrial sources of this substrate for dentifrices comprise such H_2_O_2_-generating enzyme systems, as glucose oxidase (GOx EC.1.1.3.4), lactose oxidase [87], and xanthine oxidase (XO, EC.1.17.3.2) [18,88].

### 5.2. Thiocyanates, Iodides, Bromides

Thiocyanate ions are the most important substrate for lactoperoxidase in terms of defensive properties, and are present in all mammalian body fluids, including plasma, milk, saliva, tears, respiratory epithelial fluid, and sweat [89,90,91]. The highest SCN^−^ concentration is recorded in saliva (0.3-3 mM), which is the result of active secretion of these ions [90,92,93,94]. 

These ions are released by the action of myrosinase on glucosinolates provided to the body mainly with Brassica vegetables such as cabbage, brussels sprouts, broccoli, kale, kohlrabi, and Chinese cabbage [95].

Thiocyanates migrate from plasma to epithelial gland cells through sodium/iodide symporters (NIS), and then to glandular secretion through cystic fibrosis conductance regulator channels (CFTR) [96,97], anoctamin-1 (ANO1, also called transmembrane member 16A, TMEM16A) belonging to calcium-activated chloride channels (CaCC) [98] or pendrins [99].

Besides thiocyanates, iodides, and bromides are other substrates for salivary peroxidase, however playing a less important role in microbial defense due to their low physiological concentration. In contrast to SCN^−^ whose industrial source is potassium thiocyanate [100,101], these substrates are not used in industry as an additive to oral care products.

## 6. Chemical Mechanism of Action

The first stage in the cycle of lactoperoxidase activity is the transition from the native form to Compound I by the two-electron oxidation in the presence of hydrogen peroxide (alternatively, organic peroxides [102]), which is reduced to water [18]. 

The creation of Compound I can be represented by the reaction

LPO (native form) + H_2_O_2_ → LPO (Compound I) + H_2_O

### 6.1. Halogenation Cycle

LPO Compound I can enter the cycle of halogenation in the presence of two-electron donors such as SCN^−^, I^−^, and Br^−^. It passes through a one-step two-electron reduction of Compound I to the native form. (Pseudo)halides (I^−^, Br^−^, and SCN^−^), in turn, oxidize to hypo(pseudo)halides (OI^−^, OBr^−^, and OSCN^−^) giving up two electrons [2,103]. SCN^−^ is the most preferable ion for oxidizing under physiological conditions, considering that its highest concentration is associated with high supply with food, active secretion into the saliva and the highest value of the reaction rate constant [104,105].

In summary, the reaction of the halogenation cycle can be represented as follows:

LPO (Compound I) + X^−^ → LPO (native form) + OX^−^

### 6.2. Peroxidation Cycle

The peroxidation cycle consists of a two-electron reduction to the native enzyme form with the formation of a Compound II intermediate [2]. The literature describes a large number of substrates (both endogenous and exogenous) involved in this cycle [65,106,107,108]. The second stage of reduction is described as a step limiting the speed of lactoperoxidase system due to the relatively long recovery time to the native form. It is a disadvantageous step from the point of view of antimicrobial activity, because of competing with the halogenation cycle, where microbial-reactive products are produced [2].

The reactions of the peroxidation cycle can be presented as follows

LPO (Compound I) + AH → LPO (Compound II) + A^•^
LPO (Compound II) + AH → LPO (native form) + A^•^

### 6.3. Inactive Forms

Enzymatically inactive LPO Compound III is formed in the case of a molar excess of H_2_O_2_ (which may occur in the course of oral inflammatory diseases) and in the absence of single-electron donors or in reaction with hydroperoxyl radical [109]. Compound III may to a certain point undergo partial reactivation to Compound II [109,110]; however, it comes to an irreversible inactivation after a longer period of high H_2_O_2_ concentrations, through damage to heme and the release of iron.From an industrial point of view, selecting the ratio of lactoperoxidase and H2O2-generating system concentrations is an important aspect of designing preparations with LPO system in order to minimize the risk of inactive lactoperoxidase. It may also be important to use additions of LPO reactivators to prevent the formation of inactive forms and the entry of LPO into the peroxidation cycle at elevated H2O2 concentration in oral pathologies [29].

### 6.4. Reactivators

In recent years, a large number of substances have been described to react with LPO Compound II, reducing it to the native form. This action ensures rapid regeneration of the inactive (in term of reactive products formation) Compound II and prevents the formation of Compound III and constant enzyme inactivation. 

The most effective reactivators are those substances that show a high value of constant of reaction with Compound II, thus acting only as reactivators of this form [111]. At present, while there are neither in vitro studies in microbiological models nor clinical trials, only studies of the molecular mechanism of interaction of these substances with LPO; one can assume that this mechanism may constitute a new fixture point in the prevention of caries [112]. 

Reactivators include many substances of natural origin belonging to flavonoids [111], polyphenols [29], tannins [77], and cinnamic acid derivatives [112]; moreover, there are ongoing studies on the use of plant extracts containing more than one substance with such an effect [113,114]. In addition to the direct bactericidal action, Flemmig et al. showed the reactivating properties of some components of the *Olea europaea* extract on LPO Compound II [113]. Similarly, Gau et al. demonstrated the reactivating properties of tannins and their derivatives, which also exhibit direct bactericidal and anti-inflammatory effects [77]. During the investigation of the reactivation outflow of *Leonurus cardiaca* extracts performed by Flemmig et al., the team drew attention to the need of usage of selective extraction processes to enrich extracts with selected groups of active compounds and to prevent the extraction of inhibitors [113].

### 6.5. Inhibitors

The biocidal activity of LPO can be inhibited by numerous substances acting through three main mechanisms. The first of them is the pre-enzymatic mechanism consisting in competing for access to substrates. This group of inhibitors may include physiological salivary components such as myeloperoxidase (whose concentration increases in periodontitis [115]) and catalase [116] competing for hydrogen peroxide with LPO, or uric acid present in saliva at high concentration and competing with SCN^−^ ions [117]. The second group of inhibitors are substances interacting with the enzyme molecule, e.g., cyanides, azides, or thiourea [2]. The third postenzymatic mechanism of inhibition is associated with the reduction of reactive lactoperoxidase products. This group includes compounds containing thiol groups, such as glutathione present in saliva (whose concentration increases in caries [118]), which may lead to the weakening of the LPO system. This group also includes NADH-OSCN oxidoreductase, which is responsible for the OSCN^−^ breakdown to SCN^−^ [80].

The inhibitory effect on LPO is also exerted by commonly used drugs, e.g., some indazoles [119], carbidopa [120], salicylic acid and some other phenolic acids [121], propofol [122], some antibiotics, and some corticosteroids [123]. 

## 7. Biological Role of the LPO System

### 7.1. Mechanism of Biocidal Activity

The key products of the lactoperoxidase system are hypothiocyanite ions (OSCN^−^) and hypothiocyanous acid (HOSCN) formed during the oxidation of thiocyanates [124,125]. Both products are in a dynamic equilibrium state, transforming into one another. The ratio of dissociated form (OSCN^−^) to undissociated one (HOSCN) depends on the pH of the environment [126]. Hypothiocyanites have a strong selective oxidizing activity on the thiol moieties of key enzymes in the course of glycolysis in microorganism [127,128]. The mechanism of inactivation: a transient product (R-S-SCN) is formed as a result of the thiol group (R-SH) oxidation, which then breaks down to release a hydroxythiol moiety (R-S-OH); thiocyanate ions that can be reoxidized and undergo further reactions with thiol groups [128]. This is summarized by the following reactions.

R-SH + OSCN^−^ → R-S-SCN + OH^−^

R-S-SCN + H_2_O → R-S-OH + SCN^−^ + H^+^

Nevertheless, the inhibitory effect of OSCN^−^ ions on the growth of microorganisms may be reversible if these ions are removed from the environment and there is a correspondingly high concentration of reducing agents as glutathione or NAD(P)H in the cell [128]. The presence of intracellular NADH: hypothiocyanite oxidoreductase in commensal *Streptococcus sanguinis* effectively protects them against undesirable inhibition of growth [129]. Only the prolonged duration of HOSCN activity on the cells has the effect of irreversible inhibition due to further modification of the intermediate R-S-OH and R-S-SCN products [128].

The concentration of iodides in saliva is below 1 μM, hence their physiological oxidation by LPO is marginal as compared to thiocyanates. HOI, IO^−^, as well as I_2_, I_3_^−^, and I_2_OH^−^ are the products of the reaction between I^−^ and salivary peroxidase Compound I [130,131]. These products have a broader range of activity than hypothiocyanites, as they oxidize NAD(P)H and thioether groups in addition to the thiol moieties of proteins [2,132]. In addition to the bacteriostatic effect of hypothiocyanites, the products of iodide oxidation show bactericidal and fungicidal activity. Vanden Abbeele et al. observed a positive effect of mouth rinse on the reduction of dental plaque in in vivo studies using a mouthwash containing OI^−^ [133]. Comparing the efficacy of antifungal activity on *Candida* blastophores, the survival rate in the LPO system containing thiocyanate was 56–88%, whereas it was 0–4% in the system where iodides were applied [134]. Ahariz et al. observed greater effectiveness of the iodide system in limiting the growth of *C. albicans* blastoconidial biofilms on titanium surfaces in comparison to the LPO–H_2_O_2_–SCN^−^ system [135]. OI^−^ ions have a broad biocidal activity range, including fungi and Gram-negative and Gram-positive bacteria, and show synergy with SCN^−^ ions in LPO system (stronger activity than SCN^−^ alone) [136]. Schlorke et al. presented another possible product of the LPO system—cyanogen iodide (ICN)—which is formed in the presence of both SCN^−^ and I^−^. The strong toxic effects of this product have been described, however the exact mechanism of its action on microorganisms is not known. Although physiological formation of ICN does not occur, it seems that such selection of the SCN^−^ and I^−^ ratio in dentifrices generates significant amounts of cyanogen iodide and may be important in increasing their antimicrobial efficacy [137].

### 7.2. The Effect of the LPO System on Dental Plaque

In both caries and periodontitis, the formation of plaque or biofilm is a key element in the pathomechanism of these diseases. In the course of caries, the biofilm has supragingival localization [138], but is subgingival in periodontitis [139]. The LPO properties that inhibit the formation of biofilm at each stage of its formation have been demonstrated in literature. Due to the ability of adsorption on salivary pellicle [33], the effectiveness of the LPO system has been proved in preventing adhesion of precursor cariogenic microorganisms [140]. Similar studies have also been conducted on mature biofilms for both single- and multispecies. Cawley et al. demonstrated that the LPO system together with lactoferrin, lysozyme, and bovine milk immunoglobulins have the ability to inhibit the formation of single-species *S. mutans* biofilm, whereas, in the case of multispecies biofilms (which more accurately represents biofilms occurring in vivo), its application resulted in the loss of microbial viability without the decrease in biofilm mass [141]. This fact can be explained by the ability of the LPO system to kill microorganisms, but the inability to inactivate glucosyltransferases, i.e., extracellular bacterial enzymes responsible for the synthesis of glucans building a biofilm matrix [142].

### 7.3. Inhibition of Organic Acids Production

The local effect of organic acids produced by microorganisms as a result of carbohydrate metabolism is the direct factor causing the demineralization of the tooth surface in the course of caries [138]. Tenovuo et al. demonstrated that a dental plaque treated with the LPO system ex vivo produces less acids than the plaque in the absence of this system [42]. In addition, the production of acids is inversely proportional to the concentration of hypothiocyanates [143]. The reason for this may be the ability of the LPO system to inhibit glycolysis enzymes (one of the pathways responsible for acid production) such as hexokinase, 3-phosphoglyceraldehyde dehydrogenase, aldolase, and glucose-6-phosphate dehydrogenase [128,144,145,146]. In addition, the LPO system impairs glucose transport, which is probably caused by damage to the integrity of the cell membrane GLUT transporters [147,148,149].

With decreasing pH, the concentration of the dissociated form decreases and the concentration of undissociated forms of the halogenation cycle increases. The undissociated form is characterized by stronger biocidal properties due to the easier penetration of hydrophobic cell membranes [126]. This elevates the antibacterial effectiveness in caries, when the pH of plaque microenvironment is reduced (pH< 6) [150] against saliva (pH = 6.7–7.3) due to the production of organic acids (such as lactic and acetic acid) by bacteria (*S. mutans* and *L. acidophilus*) [124,151,152].

### 7.4. Defense against Oxidative Stress

As already mentioned, the production of H_2_O_2_ takes place in the oral cavity both by microorganisms and host cells. This compound is toxic against microorganisms and host cells such as epithelial cells or mucosal fibroblasts [1]. Its micromolar concentrations may induce aldehydic DNA lesions in the Fenton reaction pathway of mitochondrial and nuclear DNA [153]. Moreover, H_2_O_2_ causes lipid peroxidation of the cell membrane [146].

The toxicity of hydrogen peroxide is much higher than that of hypothiocyanates formed in halogenation cycle due to the entry of H_2_O_2_ into the Fenton reaction and the formation of a toxic hydroxyl radical [154]. H_2_O_2_ is decomposed not only in the LPO halogenation cycle, but also in reaction with the hypothiocyanate to produce water, molecular oxygen, and thiocyanate [146].

H_2_O_2_ + OSCN^−^ → H_2_O_2_ + O_2_ + SCN^−^

Therefore, protection of the host against ROS damage is another important function of LPO.

### 7.5. Effect on Carcinogens

Saliva is the first fluid that comes into contact with food and participates in its digestion in addition to its function of carcinogens inactivation. Along with food, numerous carcinogens are supplied to the organism, e.g., benzo(a)pyrene, aflatoxins, or pyrolisates of amino acids and proteins. Nishioka et al. demonstrated that human saliva has the ability to inactivate the mutagenicity of these compounds [155]. One of the proposed mechanisms of their inactivation is oxidation in the peroxidation cycle of lactoperoxidase [156].

On the other hand, the ability of LPO to activate certain carcinogens was also identified. There are studies proving that LPO increases the ability to bind DNA while entering the peroxidase cycle with arylamine carcinogens, which is the cause of the mutagenic activity of these compounds [157,158].

## 8. Clinical Application

### 8.1. Review of Clinical Trials and In vitro Tests

Commercially available dentifrices containing lactoperoxidase have been subjected to clinical trials involving participants from various age groups with various clinical conditions, e.g., malodor [159,160], xerostomia [161], caries [3], and chronic periodontitis [4], as well as healthy subjects [101,141,162,163,164]. The usefulness of LPO preparation was also determined during pilot studies in neonates under mechanical ventilation in order to avoid ventilator-associated pneumonia (VAP) [165]. The summary of clinical trials is provided in the first part of Table 2.

In vitro studies on the effect of lactoperoxidase on oral microorganisms are being constantly published and document its effects against *Streptococcus mutans* in caries, when used separately [142,166,167,168,169,170] or in combination with lactoferrin, lysozyme, or immunoglobulins [140,141,171,172,173,174]. The ability of *S. mutans* to create biofilms have been taken into account together with the activity of glucosyltransferases responsible for the synthesis of exopolysaccharides that build the biofilm matrix [142,166,168]. It has been observed that the mouth rinse foam effectively reduces the retention of biofilms produced by salivary as well as nonpathogenic bacteria of the oral cavity [171]. In addition to *S. mutans*, studies also concentrate on other oral streptococci [141,167,170], species associated with the occurrence of periodontal disease [141,169,170] and *C. albicans* fungi [167,173]. The summary of in vitro tests is presented in the second part of Table 2.

#### 8.1.1. Caries Prevention

The studies on the anti-caries effect of the LPO system show that it is most often assessed based on the impact on *S. mutans* species, which is an important element of caries development due to the ability of biofilm formation and fermentation of dietary carbohydrates. The acquisition of these pathogens at an early age is a key element in the development of early childhood caries (ECC) in children.

Short-term studies involving children affected by ECC showed that the use of fluoride-free, fluoride and enzymatic paste caused a decrease in salivary *S. mutans* within seven days. The decrease observed in the group using the enzymatic paste was the most significant, in addition to a decrease in *L. acidophilus* amount [3]. A similar effect was observed by Jyoti et al. during a 4-week study using a different enzyme paste in children with ECC where the content of salivary *S. mutans* and Lactibacillus also significantly decreased, which was associated with an increase in the concentration of thiocyanates in saliva during the paste use [175]. Lenander-Lumicari et al. did not observe any antibacterial effect on salivary *S. mutans* and Lactobacillus in a 4-week study with the use of LPO paste in adults [176].

The short-term effect of using the paste with the LPO system is based on a significant increase in the saliva content of active proteins and compounds. Cawley at al. compared the levels of H_2_O_2_ and lysozyme found in saliva directly after brushing, showing 64% and 92% higher levels, compared to the control paste, respectively [141]. The same team had also described an increase in hypothiocyanates (above 100 μM) generated by the LPO system in vitro in the saliva of volunteers after adding to it the test paste. Lenander-Lumicari et al. observed an elevated HOSCN/OSCN^−^ level (even to about 300 μM) in saliva directly after 1 min of brushing (maintained up to 20 min) [176].

Lactoperoxidase affects the enzymes involved in the synthesis of EPS that build a biofilm matrix, in addition to activity against glycolytic enzymes. Korpela et al. showed that lactoperoxidase showed an inhibitory effect on all glucosyltransferases in a certain concentration range in the absence of substrates. The activity of adsorbed D and C transferases in the LPO concentration range of 25 to 50 μg/mL was most strongly inhibited. In the case of a liquid phase, B and C transferases were inhibited to the greatest extent in the presence of LPO in a concentration range of 10 to 100 μg/mL [142]. It has been hypothesized that this phenomenon is associated with the attachment of LPO (in a sufficiently high concentration) to Gtfs, changing their conformations and consequently reducing their activity. A similar inhibitory effect on the enzyme itself without added substrates was previously noted by Yu et al. investigating the effect of LPO on GtfD derived from *S. mutans*, while the complete system strengthened GtfD activity [168]. Liu et al. observed that the addition of iodides to the LPO system limits adhesion as well as glucosyltransferase-associated EPS synthesis more efficiently than the LPO system with SCN^−^ alone. This effect increases with the increase of I^−^ concentration [166]. The effect on *S. mutans* adhesion was also described by Roger et al. where the LPO used at a concentration range of 20 to 100 μg/mL reduced the bacterial adhesion to hydroxyapatite proportionally with enzyme concentration [140]. The LPO system effectively limits the growth of *C. albicans* fungi in addition to *S. mutans*. Welk et al. used a quantitative suspension test for *S. mutans*, *S. sanguinis*, and *C. albicans* in the presence of the LPO system, and demonstrated a complete killing of *S. mutans* cells after 5 min (RF 7.49) and *C. albicans* cells after 3 min (RF 6.78). The complete killing of *S. sanguinis* cells occurred after 15 min (RF 8.12) [167], which could be related to the presence of NADH: hypothiocyanite oxidoreductase in these cells protected them against OSCN^−^ [133].

The combination of lactoferrin, lysozyme, and LPO present in the toothpastes, effectively increases the permeability of *S. mutans* membrane (at a concentration of 5.2 μg/mL paste proteins), inhibits planktonic growth (at toothpaste protein concentration of 1.4 mg/mL) and reduces the viability of a single-species biofilm of this pathogen [141]. Such a combination of antibacterial proteins also worked well in the studies of Pinherio et al., who applied the concentration of 10 μg/mL of each protein and, after three applications, described a significant *S. mutans* reduction on the surface of artificially formed carious lesions on extracted teeth [172].

These studies show that the anti-caries effect of the LPO system tested in vitro, including the reduction of adhesion, biofilm viability, and inhibition of *S. mutans* growth, is reflected in the results of in vivo studies. Oral care products used in them, work together with LPO thanks to the addition of active proteins such as lactoferrin and lysozyme, intensifying their action especially during teeth brushing, when the content of active ingredients in the oral cavity is the highest.

#### 8.1.2. Periodontal Disease

Regular and effective removal of dental plaque accumulating in the subgingival zone is an important element of prevention of gum and periodontal diseases. Such accumulation results in the development of the inflammatory response of the host with the progressive destruction of the tissues surrounding the tooth [139]. Numerous studies in the literature described anaerobic bacteria associated with these diseases, e.g., *P. gingivalis* [177,178], *A. actinomycetemcomitans* [179,180], *F. nucleatum* [181], and *T. denticola* [182].

The results of the studies presented in Table 2 demonstrate that the use of lozenges containing LPO, LF, and GOx gave inconclusive results in terms of the effect on bacteria associated with periodontitis. Shimizu et al. described that the effect on total bacterial count and *P. gingivalis* number (in saliva and supragingival plaque) of people with chronic periodontitis was not significantly different from the control after 12 weeks of tablet (LPO 1.8 mg/tab) administration three times a day [4]. Morita et al. observed the reduction in the number of *P. gingivalis* and *F. nucleatum* colonizing the tongue as well as the restriction of tongue coating in the control and test group after eight weeks of tablet usage (LPO 2.6 mg/tab) [163]. These results may be related not necessarily to the antibacterial effect of LPO together with LF, but with the mechanical removal of the coating during tablet suction. Tablets, although having a system ensuring H_2_O_2_ generation (i.e., Gox + glucose) and a very high LPO concentration, did not provide SCN^−^ ions unlike the pastes, hence saliva was their sole source.

Enzymatic toothpastes are frequently used in clinical trials in addition to tablets. Daly et al. observed that the use of enzymatic paste in healthy subjects for 13 weeks significantly affected such parameters of gingival health, as modified gingival index, bleeding index, and plaque index, as compared to the control group using fluoride-only paste [164]. The same paste showed efficacy in increasing the permeability of the cell membrane (in the presence of 7.8 μg/mL paste proteins) and inhibiting the planktonic growth of *F. nucleatum* (in the presence of 1.4 mg/mL paste proteins). Tenovuo et al. showed that *B. cereus*, i.e., another periodontopathic species, shows suppression of planktonic growth in the presence of the LPO system (in 2 μg/mL concentration). This effect escalated with the amount of generated OSCN^−^, reaching 100% for 169 μM of this product. Paste dilutions used by Cawley et al. were similar to that occurring in oral cavity during brushing and showed a reduction in the viability of a seven-species biofilm composed of commensal and pathogenic bacteria associated with periodontal disease (Table 2). Wikström et al. examineda reduction in visible plaque score, which was obtained in the study group within 12 months of a weekly professional tooth cleaning with LPO paste. Viable microbial count of *F. nucleatum* in the tongue coating and supragingival plaque was lower than in the control group. The proportion of Lactobacilli in the dental plaque in both groups during the experiment, however, gradually increased in supragingival plaque while there were no significant differences in the microbial count of *S. mutans*, *Lactobacillus* spp., and *Actinomyces* between the control and test groups [162]. This could be due to the simultaneous intake of several drugs by the participants and the associated reduction in salivary flow rate (<4.0 μL/cm/min in 76% of participants), which resulted in decreased buffering capacity of the organic acids produced by acidogenic bacteria, creating a favorable environment for their growth.

It appears that the composition of the preparation ensuring adequate access of substrates for lactoperoxidase is an important factor in the products used for oral hygiene. Tooth brushing allows one to remove dental plaque and assure an access of active proteins to the deeper layers of biofilm. This provides a noticeable reduction of visible plaque as well as improved gum health.

#### 8.1.3. Other Applications

The study conducted by Nakano et al. with the use of the tablets with LPO (2.6 mg/tab) and LF in patients with malodor report a significant reduction in the level of VSCs and H_2_S within 10 min after a single administration compared to control [159]. After the same time after administration of a tablet containing 1.8 mg of LPO per tablet, Shin et al. observed a decrease in CH_3_SH and VSCs compared to the control [160]. This positive effect on the reduction of malodor can be associated with the mechanical removal of the tongue coating (which has a significant impact on the level of VSCs [184,185]) on the one hand, and with the possible OSCN^−^/HOSCN activity produced by LPO, on the other. These products react with the cysteine residues of the methionine γ-lyase enzyme present in anaerobic periodontopathic bacteria (e.g., *F. nucleatum* and *P. gingivalis*) and inhibit its activity and the formation of VSCs responsible for bad breath [186].

Xerostomia is another oral disease, where LPO-containing products are used. The protective effect of saliva in patients struggling with this problem is limited, which results in their greater susceptibility to the development of caries [187]. There are numerous products in the form of gels and rinses that contain natural defensive proteins, including LPO, LYS, LF, and immunoglobulins [188]. These products effectively relieve symptoms such as swallowing problems, dry mouth, loss of taste [161]. Guneri et al. showed in vitro inhibitory effect for only some moisturizing products on the growth of *S. mutans* and *L. acidophilus* and no inhibition of *C. albicans* growth. Nevertheless, due to the lack of data on the exact composition of the products, the question what could cause these discrepancies was not resolved [188]. Silva et al. did not observe any inhibitory effect artificial saliva containing LPO, LF, and LYS on the formation of *C. albicans* biofilm on acrylic plaques in contrast to saliva containing carboxymethylcellulose (CMC) [173]. A 2-week clinical trial in patients suffering from xerostomia after irradiation didn’t show any significant differences in the amount of *S. mutans*, *L. acidophilus*, and *C. albicans* present in the saliva between the therapy using products containing LPO, LYS, and LF, or the one containing CMC [189].

Stefanescu et al. carried out pilot studies to pre-determine whether a regular application of an oral hygiene gel containing LPO, LYS, and LF would allow the prevention of VAP in mechanically ventilated infants. The tracheal flora samples collected during the study showed no differences in composition, density, or changes of density compared to the control group. There were no differences in the time of hospitalization or the time remaining under mechanical ventilation. It was suggested that the results could have been influenced by the late intervention onset (after the 7^th^ day of life), endotracheal tube, and a previous use of antibiotics in some infants [165].

### 8.2. Dentifrices with the LPO System

Available dentifrices containing the LPO system usually consist of lactoperoxidase, potassium thiocyanate, and a system for generating hydrogen peroxide such as glucose oxidase (GOx), xanthine oxidase (XO), or lactose oxidase. In addition, these preparations are often enriched with other nonspecific bioactive components such as lactoferrin, lysozyme, or antigen-specific immunoglobulins of bovine milk.

Lactoferrin is a glycoprotein belonging to the transferrin family that has the ability to bind iron. Its antibacterial properties are due to the ability of iron sequestration which makes it inaccessible to many species of bacteria, including cariogenic *S. mutans* [190]. In addition, it interacts with Gram-negative lipopolysaccharide, releasing it into the environment [191].

Lysozyme (EC.3.2.1.17), or muramidase, is a strongly cationic antibacterial protein found in many body fluids [192]. Its activity is based on the hydrolysis of glycosidic bonds in peptidoglycan of bacterial cell walls [193,194] and increase in cell membrane permeability, as well as inhibiting biofilm formation [195].

The combination of naturally occurring proteins found in oral hygiene products enhances their mutual effects and supports the antimicrobial protection system of the host, resulting in improved gingival health parameters and changes in ecology of the bacterial flora, i.e., the increase of the share of species related to oral health [183]. Protein components of the pastes may be deposited on the surface of the renewing pellicle after brushing. In the case of LPO, its activity was detectable after 40 min from the formation of the acquired pellicle for three tested enzymatic pastes [196]. Despite the market availability of many other forms of application of oral hygiene products containing LPO (such as lozenges, gels, foams, and mouthwashes), it is important to remember that they constitute a supplement to teeth brushing and can be easily omitted during an everyday oral care routine, while providing a convenient alternative to a toothpaste for use outside the home [171].

Currently, caries prophylaxis involves the use of classic fluoride preparations [187,197,198] and intensively studied nonfluoride methods including chlorhexidine [199], probiotics [200], xylitol [199,201], triclosan [202], CPP-ACP [203], and proteins of natural origin. Dentifrices with the addition of antibacterial proteins such as LPO may be an alternative to common fluoride preparations.

The currently recommended form of caries prophylaxis involves the use of pastes and other products containing fluoride [204]. Despite the wide prevalence of this prophylaxis, caries and periodontal disease are still a major problem in every age group. The use of varnishes and gels with a high content of F^−^ and low pH (3.5–5) may cause local inhibition of OSCN^−^ production through the LPO system, whereas fluoride present in enzymatic pastes does not show any inhibitory effect on the LPO system because it ensures pH > 5.5 [205].

A mild surfactant, stearyl ethoxylate 30, is used in pastes with the LPO system instead of the commonly used sodium lauryl sulfate (SDS). SLS has been shown to cause reduction of the protective effect of mucin of the mucous membrane, desquamation of oral epithelium, and contributes to the development of irritation [206,207] and gingival sloughing [206].

Chlorhexidine (CHX) used in rinses and gels as one of the ways to reduce plaque in gingivitis, is characterized by a strong bactericidal effect due to its positive charge allowing adsorption on the mucous surface, teeth, biofilm components including bacteria, EPS, and glycoproteins [208]. The long-term use of this agent is, however, associated with the occurrence of discoloration of teeth [209,210], tongue and mucous membrane [208], taste loss [211], epithelial desquamation, and calculus formation [212]. In contrast to chlorhexidine, the products of the LPO system have a selective effect (does not show biocidal effects on certain species of microbes of physiological flora) and do not damage the cells of the host [133]. The effect of discoloration was also described after using a rinse containing stannous fluoride (SnF_2_) and essential oils [213]. CPP-ACP is used in the remineralization of enamel but has no antibacterial activity compared to LPO [172]. On the other hand, the habitual consumption of xylitol showing bacteriostatic and antibiofilm activity [214], may lead to the growth of xylitol-resistant *S. mutans* strains in the plaque and loss of its effectiveness [215]. So far, no side effects have been demonstrated due to the long-term use of preparations containing lactoperoxidase.

## 9. Stability Extension

Due to the widespread use of lactoperoxidase in the cosmetic, pharmaceutical, and food industries, it is required to increase its stability in finished products and ensure a long period of its activity maintenance. The choice of stabilizing substances is very important in creation of oral care preparations containing active enzymes in an aqueous environment, so that they do not degrade during a long-term storage at a room temperature. Nimatullah et al. showed that LPO stored in the aqueous environment quickly loses its activity. They demonstrated a total loss of its activity at 25 °C during the first week, while at 4 °C the sample lost half its initial activity during the third week of the experiment. The best effects have been proven in the case of −20 °C freezing, as there was no activity decrease throughout the duration of the 4-week experiment [216]. As it is practical for oral hygiene preparations to be stable at room temperature, methods to ensure this are still being sought.

Lyophilization (freeze-drying) is one of the most effective processes for prolonging the stability of biologically active compounds. In addition, lyophilized substances take up considerably less space during storage than solutions [217]. Shariat et al. showed that the use of lyophilization with trehalose as a lyoprotectant allowed LPO to be fully active for 41 days (duration of the experiment), while the freeze-dried enzyme without the addition of trehalose lost its activity below 5% after 2 days [218]. This method of stability extension is well suited for preparations for use in the laboratory or industry, but is not suitable for oral care preparations for practical reasons.

Osmolytes are substances that protect proteins against loss of their function due to the action of temperature, urea or salt [219]. Boroujeni et al. also examined the possibility of using osmolyte ectoine as a stabilizing agent for the changes in pH and temperature [220]. The enzyme remained the most active at 25 °C and pH of 6.4 in the presence of ectoine. The change in pH resulted in a decrease in LPO activity; however, it did not decrease below approximately 50% of the initial value. With the pH maintained at the 6.4 level, and the temperature increased from 25 to 70 °C, the addition of ectoine allowed for the maintaining of over 80% LPO activity [220]. In the context of oral care preparations, the use of ectoine as an LPO stabilizer is safe due to the lack of toxicity of this compound [221] and the assurance of stabilization in a water environment such as toothpaste.

Immobilization on solid particles as polymers or nanoparticles is more and more widely used technique of enzyme stabilization. Jafar et al. demonstrated that the immobilization of LPO on polyaniline molecules allowed full enzyme activity at 4 °C for 60 days, while the native LPO sample lost 80% of its original activity. In addition, immobilized LPO showed a greater stability to the pH of the environment and was more difficult to denature at 60 °C [222].

### Application of Nanotechnology

Nanoparticles are characterized by a high surface to volume ratio, therefore different physical and chemical properties than the macroforms of the same compound/element [223]. Due to their properties, they are used as drug carriers allowing easier penetration into the cells, increasing the durability of the drug and controlling its release; moreover, showing a therapeutic effect in some cases [224].

Altinkaynak et al. combined hybrid copper nanoflowers with LPO which contributed to the extension of the preparation’s durability. Researchers showed that only 5% of the initial LPO activity was lost at 20 °C after 15 days using this combination, as compared to 95% loss of activity of the native LPO preparation under the same conditions. In most cases, the adsorption of enzymes on nanoparticles contributed to a decrease in their specific activity, however, in this case the combination of lactoperoxidase with hybrid copper nanoflowers increased this enzymatic parameter [225]. A similar increase in enzyme activity using silver nanoparticle conjugates (AgNPs) was obtained by Sheikh et al. [226]. Such immobilization of LPO probably allowed for increasing the stability of the enzyme with an additional increase in the surface where SCN^−^ ions could be oxidized. This resulted in an evident inhibition of *E. coli* growth just after 2 h. AgNPs have also demonstrated stabilizing properties on the enzyme, because the bactericidal action of LPO in combination with AgNPs was maintained throughout the duration of the experiment, in contrast to the native enzyme preparation with a loss in this property after 6 h [226]. In addition, AgNPs themselves have antimicrobial properties, further enhancing the biocidal effect of the conjugate [227], which has been used in the prevention of caries by creating toothpastes containing AgNPs [228].

Another example of nanoparticles use with LPO is described by Shariat et al. who performed immobilization on graphene oxide nanosheets (GO-NS) [229]. It resulted in increased LPO activity at a more basic pH compared to the enzyme itself, but was also maintained at high activity at 70 °C. Additionally, the immobilized LPO maintained activity at the level of 64% of the initial value after 30 days of storage [229].

These examples demonstrate the additional advantages of using particular stabilizers for LPO, especially in terms of potential use in oral preparations that must be stable when stored for a long time at a room temperature. Moreover, the use of nanomaterials may have additional benefits in the form of their own biocidal activity against microorganisms responsible for oral infectious diseases.

There are still few studies on the use of nanomaterials to stabilize LPO, moreover, the exact mechanism of this stabilization and the increase in specific enzyme activity observed by some teams of researchers [225,226] is unknown. One of the proposed theories of protein stabilization by nanoparticles is the steric repulsion between proteins deposited on nanoparticles, which prevents their aggregation [230]. Determination of the toxicological safety of nanomaterials in relation to humans and the environment is another clinically important aspect [231].

## 10. Summary

In recent years, intensive research has been carried out on nonfluoride methods of caries prevention as well as new methods of alleviating the symptoms of periodontitis. Compounds of natural origin such as lactoperoxidase are of particular interest. This paper presents the results of a series of studies proving the effectiveness of the lactoperoxidase system in the prevention and alleviation of the symptoms of particular oral diseases. It seems that the next step in the research on the LPO potential will be aimed at finding other reactivators of this enzyme as well as determining their effectiveness in microbiological systems and then assessing their usefulness in clinical trials. The promising field of research comprises the use of nanotechnology in the creation of new carriers of lactoperoxidase that would favorably affect its properties.

## Figures and Tables

**Table 1:** Structure and physiochemical properties of human and bovine lactoperoxidase.

Characteristic	Value	Reference
Mass	~80 000 Da (hLPO); ~78 000 Da (bLPO)	[10,18]
Gene-containing chromosome	17 (hLPO); 19 (bLPO)	[7,20]
Number of amino acid residues	632 (hLPO); 612 (bLPO)	[18]
Number of glycosylation sites	5 (hLPO); 4 (bLPO)	[14]
Isoelectric point	9.6	[18]
Optimal conditions	50 °C; pH = 6	[5]
Stability of the secondary structure	Start of degradation at 65 °C; Tm = 71.2 °C	[15]
Stability of heme	Start of degradation at 70 °C; Tm = 73 °C; Degradation at pH < 4	[15]

**Table 2:** Summary of clinical trials and in vitro tests using lactoperoxidase.

Clinical Trials				
Preparation	Test Group	Tested Parameter	Effect	References
Biotène^®^ Dry Mouth Moisturizing Spray (lysozyme, lactoferrin, LPO)	During the recruitment of people suffering from dry mouth syndrome	Time after which the participant will have a dry mouth feeling	The study is ongoing	Clinicaltrials.gov NCT03663231
Zendium^TM^ (amyloglucosidase, *GOx*, LPO)	229 healthy subjects Mean age = 32.6 years	Modified Gingival Index (MGI) Bleeding Index (BI) Plaque Index	Significant reduction vs. MGI (1.627 vs. 1.404) and PI (2.233 vs. 2.112) baseline levels in test group; significant reduction of all parameters compared to the control group	Daly et al. 2019 [164]
Zendium^TM^ (amyloglucosidase, *GOx*, LPO, *LYS*, *LF*)	46 healthy subjects Mean age = 42 years	H_2_O_2_ and lysozyme levels after brushing	64% higher H_2_O_2_ and 92% higher lysozyme concentrations vs. concentrations after brushing with a control paste	Cawley et al. 2019 [141]
Zendium^TM^ (amyloglucosidase, *GOx*, LPO)	115 healthy subjects Mean age = 42 years	Composition of supra-gingival dental plaque expressed as a mean relative abundance (MRA)	In test group: significant changes in MRA of 37 taxa; the highest MRA increase in *Neisseria flava* (2.9%), *K. denitrificans* (0.7%), *P. melaninogenica* (0.4%); the lowest MRA decrease in *R. dentocariosa* (3.2%), Bacteroidales (0.2%), *Treponema* spp (0.08%)	Adams et al. 2017 [183]
Zendium^TM^ (amyloglucosidase, *GOx*, LPO)	68 patients Age 83.7 ± 7.4 years	Composition of oral microflora collected from supra-gingival and lingual area, the presence of visible supra-gingival plaque (SP)	Significant plaque score reduction after 12 months in test group vs. baseline level (1.7 ± 0.5 vs. 0.7 ± 0.5) and vs. control (1.6 ± 0.4 vs. 1.6 ± 0.6); lack or thin layer of plaque after 12 months in 92% of test group; *F. nucleatum* count and ration significantly decreased in test group; significant decreased of *S. sanguinis/oralis* count during 12 months	Wikström et al. 2017 [162]
Orabarrier^TM^ (lactoferrin, LPO, *GOx*)	47 healthy subjects Mean age (test group) = 80.4 ± 6.4 years (control group) = 85.9 ± 6.7 years	Plaque control record (PCR), probing, pocket depth (PPD), bleeding on probing (BOP), tongue coating score, volatile sulfur compounds (VSCs), H_2_S, CH_3_SH, mouth dryness, Composition of oral microflora collected from supra-gingival and lingual area	Significant PPD (after 8 weeks) and BOP (after 8 weeks in test group and after 4 weeks in controls) decrease in both groups, tongue coating score decrease after 4 and 8 weeks in both groups; decrease in *P. gingivalis* and *F. nucleatum* count after 8 weeks in test group	Morita et al. 2017 [163]
Orabarrier^TM^ (lactoferrin, **LPO**, *GOx*)	40 participants with VSCs exceeding the olfactory threshold in the expired air Age 49.4 ± 15.3 years	Volatile sulfur compounds (VSCs), H_2_S, CH_3_SH in exhaled air 10 and 30 min after tablet administration	VSCs and H_2_S decrease (57% and 45%, respectively); no significant changes of CH_3_SH concentration between groups after 10 and 30 min; significantly lower VSCs (0.115 ± 0.078) and H_2_S (−0.085 ± 0.083) concentrations in test group after 10 min vs. controls	Nakano et al. 2016 [159]
Bioxtra^®^ (GOx, **LPO**, lactoferrin)	30 children with severe early childhood caries Age 3–5 years	Salivary *S. mutans i L. acidophilus* count (CFU) before, right after and after 7 days of toothpaste usage	Significant decrease in CFU of *S. mutans* (112.0 ± 13.3 vs. 8.50 ± 5.89) and *L. acidophilus* (109.6 ± 14.3 vs. 8.60 ± 3.98) in an enzymatic paste group during 7 days; significant decrease of *S. mutans* count in fluoride (123.1 ± 21.1 vs. 33.6 ± 10.3) and nonfluoride (110 ± 12.4 vs. 76.2 ± 19.6) paste groups	Gudipaneni et al. 2014 [3]
Biotene OralBalance^®^ gel (**LPO**, lysozyme lactoferrin)	41 mechanically ventilated newborns Age 7–10 days	Respiratory outcomes, non-respiratory short-term outcomes, time of ventilation, composition tracheal aspirate samples	No significant differences in the duration of mechanical ventilation; significantly longer hospitalization of newborns in the study group; no significant differences in the composition of the tracheal bacterial flora	Stefanescu et al. 2013 [165]
Orabarrier^TM^ Tablets (lactoferrin, **LPO**, *GOx*)	74 subjects with chronic periodontitis Age 32–73 years	Effect on human and bovine LF level, *P. gingivalis* count, exotoxin level, total bacterial count in saliva and gingival fluid, plaque control record (PCR), probing, depth (PD), bleeding on probing (BOP), plaque index (PI), gingival index (GI), CAL (clinical attachment level)	Significantly higher bLF concentration in saliva and CGF in study group vs. controls; non-significant effect on bacterial parameters; no significant differences in periodontal health parameters vs. control group	Shimizu et al. 2011 [4]
Orabarrier^TM^ (lactoferrin, **LPO**, GOx)	15 participants with VSCs exceeding the olfactory threshold in the expired air	Volatile sulfur compounds (VSCs), CH_3_SH in exhaled air 10 min, 1 h and 2 h after tablet administration, bacterial count in saliva	No significant differences in salivary bacterial count; significantly lower CH_3_SH concentration in test group vs. controls	Shin et al. 2011 [160]
BioXtra^®^ (GOx, **LPO**, lactoferrin)	34 patients with radiotherapy-induced xerostomia Age 63.5 ± 9.4 years	Intensity of xerostomia symptoms, effect on dysphagia, pain in oral cavity, loss of taste (0–3 scale)	Remission of symptoms severity, a significant reduction in dry mouth feeling (2.03 vs. 1.12), dysphagia improvement (1.62 vs. 0.76) after 28 days	Dirix et al. 2007 [161]
Biotene^®^ (GOx, **LPO**)	12 healthy subjects	SCN^−^, HOSCN/OSCN right after brushing	Increase in salivary HOSCN/OSCN-Level and its decomposition after 20 min	Lenander-Lumikari et al. 1993 [176]
Biotene^®^ (GOx, **LPO**)	26 healthy subjects	SCN^−^, HOSCN/OSCN, total bacterial count in saliva, streptococcal count, *S. mutans*, *Lactobacillus* spp	No significant inhibitory effect on the growth of the tested species	Lenander-Lumikari et al. 1993 [176]
**In vitro studies**				
**Tested substance**	**Microorganism**	**Tested parameter**	**Effect**	**References**
Amyloglucosidase, GOx, **LPO**, LF, LYS and bovine colostrum (IgG)	*S. mutans*, *Fusobacterium nucleatum*	Film integrity and polarity using fluorescent dyes	Significant increase in fluorescence connected to a film polarity (33.3% for *S. mutans*) and permeability (44.4% increase for *S. mutans* and 57.6% increase for *F. nucleatum*)	Cawley et al. 2019 [141]
Amyloglucosidase, GOx, **LPO**, LF, LYS and bovine colostrum (IgG)	*S. mutans* *Fusobacterium nucleatum*	Effect of toothpaste on planktonic growth	Significant reduction in the growth of both strains	Cawley et al. 2019 [141]
Zendium^TM^	*S. mutans* *Fusobacterium nucleatum*	Effect of toothpaste on the viability of a single-species biofilm	Significant reduction in *S. mutans* biofilm viability (40% decrease in fluorescence); insignificant reduction of *F. nucleatum* biofilm viability (23% decrease in fluorescence) vs. control paste	Cawley et al. 2019 [141]
Zendium^TM^	*S. mitis* *S. intermedius* *S. oralis* *Actinomyces naeslundii* *Veillonella dispar* *Fusobacterium nucleatum* *Prevotella intermedia*	Effect of toothpaste on the viability of a seven-species biofilm	30% decrease of viability after 2 h; 27% decrease of viability after 4 h; 47% decrease of viability after 8h vs. control paste	Cawley et al. 2019 [141]
Splat Oral Care Foam (LF, GOx, **LPO**)	*Staphylococcus aureus* *Kocuria rhizophila* *Micrococcus thailandicus*, *E. coli* *Chromobacterium violaceum* bacteria from pooled saliva	Retention test of growing and mature biofilms made on glass, Teflon and tooth surface after 5 and 30 s of rinsing with foam	Reduction of biofilm retention on glass and Teflon after foam rinsing for 30 s (all species except *C. violaceum*; 86.9% retention on Teflon); significant reduction of biofilm retention from pooled saliva on enamel	Jones et al. 2018 [171]
MPO, CAT, **LPO**, HRP	*S. sanguinis*, *S. cristatus*, *S. gordonii*, *S. parasanguinis*, *S. mitis*, *S. salivarius*, *S. oralis*, *A. viscosus*, *S. mutans*, *Actinomyces naeslundii*, *Veilonella parvula*, *S. sorbinus*, *Prevotella intermedia*, *Porphyromonas gingivalis*, *Fusobacterium nucleatum*, *Aggregatibacter*, *actinomycetemcomitans*	Evaluation of multispecies ecology in terms of the inhibitory effect of peroxidases at the concentration of those in saliva, gingival fluid present in PD and gums, on the inhibitory effects of commensal bacteria on pathogenic strains	MPO, CAT, and HRP reduce the inhibitory effect of commensal biofilms in concentrations occurring in the gingival fluid in patients with periodontitis and gingivitis; higher *P. gingivalis* and *P. intermedia* overgrowth in a multispecies biofilm compared to the increase of CF myeloperoxidase in healthy subjects; the presence of LPO and MPO in salivary concentrations found in people with periodontitis resulted in *P. gingivalis* and *P. intermedia* overgrowth; LPO had an inhibitory effect on planktonic growth of *A. naeslundii*, *A. viscosus* and growth of *S. sobrinus* and *S. oralis* in biofilms	Herrero et al. 2018 [170]
(1) **LPO**, LF, LYS (LLL); (2) casein phosphopeptide-amorphous calcium phosphate (CPP-ACP)	*S. mutans*	Bacterial count in induced carious lesions on human teeth	Significant reduction of *S. mutans* count after 3-fold LLL administration; CPP-ACP does not reduce *S. mutans* count	Pinherio et al. 2017 [172]
**LPO** system, iodide	*S. mutans*	Measurement of the effect on growth, glucosyltransferase activity, adhesion and synthesis of exopolysaccharides (EPS)	The inhibitory effect of the system increases with I^−^ concentration; reduction of glucosyltransferase activity and EPS synthesis	Liu et al. 2014 [166]
2 artificial salivae containing (1) GOx, LF, LYS, **LPO** (2) carboxymethylcellulose	*Candida albicans*	Comparison of the degree of inhibition of *C. albicans* growth on acrylic plates	Saliva containing enzymes did not show a statistically significant reduction in the amount of microorganisms vs. saliva with carboxymethylcellulose	Silva et al. 2012 [173]
**LPO** system	*S. mutans* *S. sanguinis* *C. albicans*	Quantitative suspension test and calculation of reduction factor (RF) after 1, 3, 5 and 15 min	Suspensions treated with only SCN/H_2_O_2_ mixtures did not show antibacterial or antifungal activity; LPO addition significantly increased RF of all microorganisms	Welk et al. 2009 [167]
**LPO** system	*S. mutans* glucosyltransferases B, C, and D	Glucosyltransferases activity in the liquid phase and adsorbed on hydroxyapatites	Significant inhibition of GtfC and GtfD adsorbed by the system; no effect on GtfC activity and an increase in GtfB activity in the liquid phase	Korpela et al. 2002 [142]
**LPO** system	*S. mutans* glucosyltransferase D	Glucosyltransferase activity	GtfD activity increased by the system; no influence of OSCN^−^ on GtfD activity; LPO inhibits GtfD activity in low concentrations	Yu et al. 2000 [168]
Immune whey **LPO** system	*S. mutans* serotype c	Inhibition of glucose retention	Exposure to HOSCN/OSCN^−^ as a product of LPO action enhances the inhibition by the immune whey glucose retention	Loimaranta et al. 1998 [174]
**LPO** LYS	*S. mutans* serotype c	Capacity of a strain to adhere to hydroxyapatite previously treated with saliva after administration of specific concentrations of the substance	Significant reduction in adhesion	Roger et al. 1994 [140]
**LPO** system	*Bacillus cereus*	Growth curves	Inhibition of bacterial growth proportional to the concentration of the produced OSCN^−^ ion	Tenovuo et al. 1985 [169]

## Abbreviations

LPO	Lactoperoxidases
SDS-PAGE	Sodium dodecyl sulfate-polyacrylamide gel electrophoresis
GOx	Glucose oxidase
LYS	Lysozyme
LF	Lactoferrin
